# Relationship between Structural Characteristics of Cellulose Nanocrystals Obtained from Kraft Pulp

**DOI:** 10.3390/nano10091775

**Published:** 2020-09-08

**Authors:** María Graciela Aguayo, Arturo Fernández-Pérez, Claudia Oviedo, Guillermo Reyes, Pablo Reyes-Contreras

**Affiliations:** 1 Centro de Biomateriales y Nanotecnología, Universidad del Bío-Bío, Concepción C.P. 4081112, Chile; 2 Facultad de Ciencias, Depto. de Física, Universidad del Bío-Bío, Concepción C.P. 4081112, Chile; arturofe@ubiobio.cl; 3 Facultad de Ciencias, Depto. de Química, Universidad del Bío-Bío, Concepción C.P. 4081112, Chile; coviedo@ubiobio.cl; 4 Biobased Colloids and Materials, Department of Bioproducts and Biosystems, School of Chemical Engineering, Aalto University, FI-00076 Espoo, Finland; guillermo.reyes@aalto.fi; 5 Centro de Excelencia en Nanotecnología Leitat Chile (CEN), LEITAT Chile, Santiago C.P. 7500000, Chile; preyes@leitat.cl

**Keywords:** cellulose nanocrystals, kraft pulp, CNC characterization, characterization techniques

## Abstract

Kraft pulp cellulose was hydrolyzed using sulfuric acid, under different thermophysical conditions of temperature, time, pulp concentration, and sonication time. The experimental design revealed the effect of these conditions and their interaction on the hydrolysis yield obtained. In addition, the top five cellulose nanocrystals (CNCs) yields from this experiment design were analyzed. The results obtained indicated that CNCs possess a morphology that can be described as individualized rod particles, with average diameters less than 50 nm and different size distribution. In the analysis of CNCs features, significant Pearson correlations were established between the crystallinity of the CNC, CNC yield, and interplanar crystallites distance (Δ*d*/*d*). The thermogravimetric (DTG) profiles exhibited two CNCs degradation stages, where the second stage CNCs degradation showed a significative correlation with CNC sulfur content. In our analysis, the crystallographic parameters exhibited a correlation with the mechanical behavior of the CNC, since the potential variation between the distances of the crystalline planes is related to the stress and deformation present in the crystallites of CNCs. This study provides new knowledge regarding CNCs, further enhancing information for CNC-based industries and the processability of CNCs for the development of new materials.

## 1. Introduction

In the development of new materials, cellulose, considered a green alternative to non-degradable fossil fuel-based polymers, has gained substantial interest due to its biodegradability, renewability, sustainability and biocompatibility [1]. Cellulose nanocrystals (CNCs) are commonly produced by the acid hydrolysis method from cellulosic fibers that are renewable and abundant. The cellulose hydrolysis by strong mineral acids, specifically sulfuric acid, gives nano-sized, rod-like particles, highly crystalline; exhibiting a negative surface charge, which makes aqueous colloidal cellulose nanocrystal suspensions highly stable. CNCs can be used as powerful building blocks for the production of high-quality, durable, lightweight, and cost-effective products for a variety of applications [2,3].

Researchers have attempted to optimize CNCs production with sulfuric acid, giving some insights into establishing key reaction parameters. Some of the hydrolysis conditions informed for different cellulose sources: sulfuric acid concentration between 44% and 65%, reaction time between 10 min and 120 min; reaction temperature between 40 °C and 80 °C and liquid–solid ratio in milliliter per gram of pulp between 8.75 and 20 [4,5,6,7,8,9,10,11]. Besides, in the search to evaluate the potential of agro-industrial waste and its integration in a circular bioeconomy as a platform for the development of new biomaterials, the study of CNCs has become a research topic. Agro-industrial waste, such as sugarcane bagasse [12,13], sisal fiber [14,15], cotton [16,17], coconut husk [18,19], kenaf [20,21], sago seed husks [22] have been used to isolate CNCs in order to evaluate their structural properties.

Hemmati et al. [23] studied CNCs production from walnut shells, describing their physicochemical attributes, such as crystallite size, crystallinity and thermal stability, among others. The results indicated that the mean diameter of the nanostructures was 130 nm, the crystallinity of the walnut shells was 49% and after acid/alkaline hydrolysis it increased to 60%, TGA analysis revealed that the thermal stability of the CNCs was lower compared to normal cellulose. A study reported by Oliveira [24], where various biological sources were analyzed (cotton linters, coconut fibers, sisal fibers, eucalyptus sawdust, pine sawdust, sugarcane bark, and sugarcane marrow) reveals variations of physicochemical properties of CNCs even under identical synthesis conditions. The author observed quantitative correlations between the parameters of the cellulose substrates and the resulting CNC; for instance, cellulose monolayer hydration is determined by both hemicelluloses content (compositional parameter) as well as cellulose crystal width (structural parameter). These results show how the nanostructural variability of cellulose can influence its solvation behavior and ion-specific interactions.

As mentioned above, notable efforts have been made to optimize sulfuric acid hydrolysis parameters in cellulosic sources with an emphasis on CNCs yield, as well as the study of various cellulose sources for CNC production. However, when CNCs are used in new materials, primarily as reinforcement materials, their structural characteristics are of great importance; even more, the specific synthesis route determines ion-specific interactions and CNC morphologies [25].

The present work contributes to the knowledge of CNCs’ characteristics and the relationship between structural features and synthesis conditions, from bleached kraft eucalyptus pulp. For this purpose, different CNC yields have been considered from experimental design, including factors such as sulfuric acid concentration, time, temperature, and mass of raw material. Five samples of CNC with higher yield were studied in more detail and correlations where established between characteristics and physicochemical properties.

## 2. Materials and Methods

### 2.1. Materials

Bleached kraft eucalyptus pulp (BKEP), specifically eucalyptus mixes (*Eucalyptus globulus* and *Eucalyptus nitens*), was kindly provided by CMPC Pulp S.A. (Nacimiento, Biobio region, Chile). The chemical composition of the fibers was 79.3% ± 1.1 glucan, 14.7% ± 0.9 xylan, 0.14% ± 0.08 lignin and 0.27% ± 0.12 ask. The physical property determined by Fiber Tester equipment (Lorentzen and Wettre, Stockholm, Sweden) was 0.76 mm fiber lengths, 18 μm fiber width, and coarseness of 7.5 mg 100 m^−1^. The pulp was disintegrated into a fiber suspensión at 5% solids concentration. Wet pulp was vacuum-dewatered, and air-dried to 8% moisture. Sulfuric acid 95–97% p.a. EMSURE ISO (Darmstadt, Hessen, Germany) was prepared into various concentrations for hydrolysis. The water used for this purpose was double-distilled (DD water).

### 2.2. CNC from Kraft Pulp

#### 2.2.1. Design of Experiments

CNC production was carried out under different initial concentrations of the pulp of cellulose (*X*_1_), sulfuric acid concentrations (*X*_2_), reaction times (*X*_3_), temperature hydrolysis (*X*_4_), and duration of sonication (*X*_5_) in order to evaluate different yields of CNC (studied response). The assays were performed according to fractional factorial resolution V design. The variable values were coded and normalized in unitary values, where −1 is defined as the lowest value of a variable, and +1 is its highest value, respectively (Table 1). From these extreme value variables, the central point (coded 0) was set and assayed in triplicate to provide an additional level for lack of fit testing and degrees of freedom for pure error estimation, due to the replication of experiments at this point [26,27]. The run order of the trials was randomized in order to prevent systematic errors. Analysis of variance (ANOVA) was used to test the regression significance and model a lack of fit through F-tests (confidence level of 95%).

#### 2.2.2. Preparations of CNCs and Yields

CNCs from BKEP were prepared by sulfuric acid hydrolysis [8,28], different grams of pulp, according to the concentration of pulp, were added to 200 mL of the acid solution. The resulting solutions were mechanically stirred under temperature and time indicated by the design of the experiment. The reaction was stopped with cold distilled water, and the reaction products were washed three times by centrifugation (10 min, 12000 rpm). The resulting suspensions were dialyzed (Spectra/Por^®^ Dialysis Membrane MWCO: 12–14 KD, New Brunswick, NJ, USA) against distilled water to constant pH. Then, the samples were sonicated in an ice water bath and centrifuged at 9000 rpm for 5 minutes; finally, the CNCs in the liquid phase (supernatant) and cellulosic solid residues (CSR) (pellets) were separated. The yield after the hydrolysis process was calculated as the percentage of the initial weight of the used kraft pulp fibers. CNCs mass yield was calculated in terms of CSR gravimetric difference between the starting dry pulp (BKEP dried as described in 2.1) and its respective CSR mass. This CNC mass yield (CNC yield, from now on) is therefore expressed as the ratio of CSR mass to the starting dry BKEP mass, in percentage). The five best values of CNC yields were further analyzed by their Chemical Oxygen Demand (COD), determined as previously described by Wang et al. [8] and by Aguayo et al. [29]. For all CNC yield calculations, Avicel PH-101^®^ (Sigma-Aldrich, St. Louis, MO, USA) cellulose was used as the standard for calibration and all further calculations. Afterward, a proper aliquot of each treated sample was taken and added in the COD reaction tubes (Spectroquant^®^ COD cell test, Darmstadt, Hessen, Germany). Those tubes were thoroughly mixed and heated in a closed system for 2 h at 148 °C in a Spectroquant^®^ 320 Thermoreactor (MERCK, Darmstadt, Hessen, Germany). We measured 600 nm absorbance in a Shimadzu UV–vis 1603 spectrophotometer (Tokyo, Japan), and COD was determined by interpolation using calibration curves with Avicel PH-101^®^ cellulose (Sigma-Aldrich, St. Louis, MO, USA).

### 2.3. Atomic Force Microscope (AFM) Image and Morphology of CNCs

The morphology for CNCs samples was examined using an atomic force microscope (NaioAFM, Nanosurf, Liestal, Switzerland). The equipment was operated in phase-contrast mode using PPP-FMAuD Gold Coated Force Modulation AFM Probes (Nanosensors, Neuchâtel, Switzerland) at a resonance frequency of 75 kHz, spring constant of 2.8 N m^−1^ and tip radius of about 7 nm. For this purpose, the original CNCs solution was diluted fivefold with deionized water and sonicated for 30 minutes. Then, one drop of the diluted solution was incubated for 3 minutes on muscovite mica substrates (Grade V1, SPI supplies, West Chester, PA, USA), and then was gently dried with nitrogen. After this procedure, the samples are ready for AFM data acquisition, where the time of each essay was 310 ms per line, and each micrograph has 1024 lines of resolution. The CNC diameters were analyzed using AFM images, to do this, the images were processed using the ImageJ software (Fiji distribution, open-source) and the number of measurements for each sample was made according to standard ISO/TR 19716: 2016.

### 2.4. Characterization Methods of CNCs

#### 2.4.1. Sulfur Content and Zeta Potential Analysis

The sulfur content in the five CNC samples was determined using inductively-coupled plasma atomic emission spectroscopy (ICP-AES) (Optima 800, PerkinElmer, Waltham, MA, USA). For each sample, 5 ml of CNC suspension were transferred to a Teflon flask with 5 ml of 70% HNO_3_ and digested at 150 °C for 30 minutes in a microwave (Ethos One, Milestone, Sorisole, Italy) [28]. The digestion result was measured in ICP-AES. All samples were measured in triplicate. 

The colloidal stability of CNC samples was assessed through the measurement of Zeta potential (*ϕz*). The Zeta potential was determined in samples with a solids concentration of 0.05% w/w (pH = 6.0, 25 °C) using a Zeta potential equipment (Malvern Panalytical, Zetasizer Nano S, UK), the zeta potential values were obtained in three runs with 32 measurements each, the final values were calculated as the average [30].

#### 2.4.2. Fourier Transform Infrared (FTIR) Spectroscopy

The FTIR spectra were acquired by using a Nicolet 380 FT-IR Spectrometer (Thermo Fisher Scientific, Hampton, NH, USA) in the transmittance mode. About 5 mg of powder of CNCs samples were dispersed in a matrix of KBr to be mixed and pressed into a pellet. The samples were analyzed in a spectral region between 4000 and 400 cm^−1^ with a 2 cm^−1^ resolution (averaged over 32 scans).

#### 2.4.3. X-ray Diffraction (XRD) Analysis

The X-ray diffraction (XRD) analysis was performed to determine the crystallinity, crystallite size, and fractional variation in the plane spacing (Δ*d*/*d*) of the CNCs obtained. The five samples were examined using WAXS (wide-angle X-ray scattering) equipment, in the X-ray diffractometer Rigaku Smartlab^®^ (Rigaku Co, Tokyo, Japan). Approximately 50 mg of dried CNCs were placed on the sample holder for each. Angular scanning was conducted from 5° to 50° with 5° min^−1^ with Cu Kα radiation (λ = 0.154 nm), and the generator worked at 45 kV and 200 mA. The Segal Crystanillity index was calculated according to Equation (1), where *I_t_* is the total intensity of the (0 0 2) peak for cellulose *I*, and *I_a_* is the amorphous intensity [31].

(1) $$CI=\frac{I_{t}-I_{a}}{I_{t}}\times 100.$$

The crystallite size, perpendicular to the lattice plane (0 0 2) cellulose *I* was calculated in Equation (2) by the Scherrer equation [32], where *K* is the Scherrer constant (0.9), *λ* is the wavelength of the X-ray radiation (0.154 nm), *β* is the full width at half maximum of the diffraction peak (in radians) and *θ* is the diffraction angle of the peak.

(2) $$\tau =\frac{K\lambda }{\beta cos\theta }.$$

The fractional variation in the plane spacing Δ*d*/*d* for the (0 0 2) planes were calculated following Equation (3), according to Cullity and Stock [33].

(3) $$\left|\frac{\Delta d}{d}\right|=\frac{\beta }{2tan\theta }.$$

#### 2.4.4. Thermogravimetric Analysis (TGA)

The thermogravimetric analysis of CNC samples was carried out on a TGA Q50 (TA Instruments, New Castle, DE, USA) under a nitrogen atmosphere, with a gas flow of 50 mL min^−1^ from 25 °C to 600 °C at a heating rate of 10 °C min^−1^. For this analysis, about 2.0 mg of powder CNC samples were used. The weight-loss rate was obtained from derivate thermogravimetric data.

## 3. Results and Discussion

### 3.1. Design of Experiments

The advantage of using screening designs is that they allow the study of a large number of factors using a limited number of trial combinations [34]. The responses from the different experiments are given in Table 2, where the results show that yields varied between 0% and 53.9%. The response variable was defined through a surface model that describes the relationship between the dependent and independent variables by regression. The corresponding polynomial for the yield of hydrolysis was obtained using multiple linear regressions, where this represents the independent variable (response), and the linear coefficient and binary interactions represent dependent variables, indicated in equation 4, where X_1_ to X_5_ are defined in 2.2.1.

Yield = 1.20 + 0.13 × [*X*_1_] − 0.25 × [*X*_2_] + 0.17 [*X*_3_] + 0.34 × [*X*_4_] + 0.18 × [*X*_5_] − 0.15 × [*X*_1_ × *X*_4_] − 0.41 × [*X*_1_ × *X*_5_] − 0.37 × [*X*_2_ × *X*_3_] − 0.37 × [*X*_2_ × *X*_4_] − 0.08 × [*X*_3_ × *X*_4_] − 0.27 × [*X*_3_ × *X*_5_] + 0.16 × [*X*_4_ × *X*_5_]. (4)

The different coefficients indicated in Equation (4) were evaluated, all variables influenced the model, and interactions between them were also observed. The temperature has the highest effect on the acid hydrolysis yield, while time and duration of sonication have a minor yet significant effect. These three factors have a positive weighting value, indicating a direct correlation with the yield of acid hydrolisis. Only the concentration of sulfuric acid shows a negative effect in the yield, as was observed in the experiments 14, 15 and 16, where, with a high concentration of sulfuric acid leads to zero, as a result of the yield, product of degradation of the cellulose. The binary interactions show the negative effects in the yield, being ([H_2_SO_4_] x time), ([H_2_SO_4_] x temperature), basically by the presence of acid in the combination. The influence of the variables was studied by analysis of variance (ANOVA) for a confidence level of 95% in accordance with the high significance polynomial model. F (one tail) and P-values for the model were 4.415 and 0.040, respectively. At the same time, the correlation coefficient (R^2^) was 0.90. Analysis of the response surface indicated that the maximal region of the yield was found close to 30 g.

**Figure 1:**
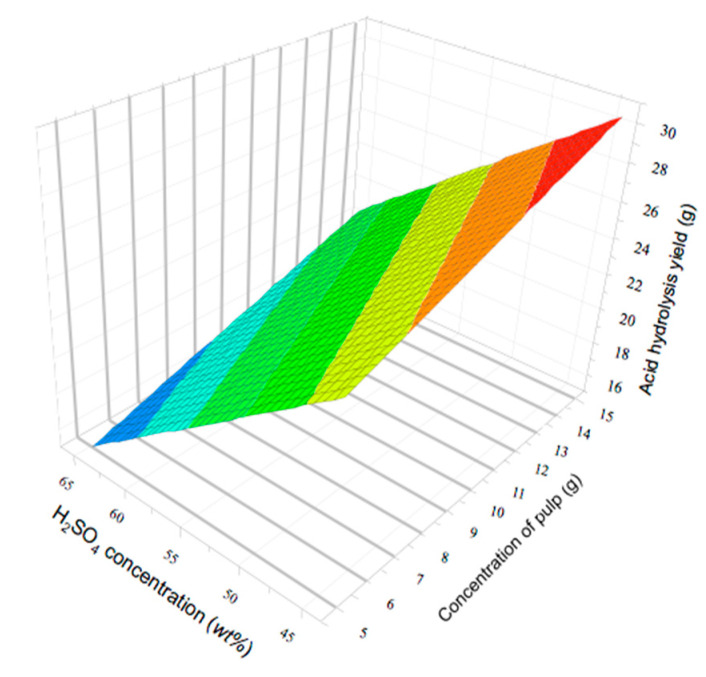
Response surface indicating acid hydrolysis yield as a function of sulfuric acid concentrations and initial concentration of pulp of cellulose. The other factors were kept constant: Time: 65 min; Temperature: 60 °C and time of sonication 20 min.

The optimal conditions showed that a similar concentration of pulp and sulfuric acid concentrations, but different time and temperature of reaction (120 min, 80 °C) and sonication (10 min), the model predicts a maximal yield of 29.9 g of solid acid hydrolysis. While that experimental value obtained under similar conditions was 28.3 g. The shape of the response surface suggests that a displacement towards higher sulfuric acid concentration and concentrations of pulp could provide even higher values, probably leading to a maximum (Figure 1).

### 3.2. Yield Analysis of CNCs

The best five CNCs yields (Table 3) obtained from the fractional factorial design were further analyzed in terms of their corresponding CNCs’ structural traits (samples: 8 = A; 10 = B; 11 = C; 13 = D and 17 = D). The best samples concerning yields were C and D, whichever the yield methodology applied. Hot sulphuric hydrolysis is an extremely harsh treatment, the supernatant phase obtained in 2.2.2., which is rich in CNCs, can hold cellulose fragmentation monomers such as glucose, or even its degradation products, as well as xylose and hydroxymethylfurfural (HMF), among others. Interestingly, Wang et al. [35], built a kinetic model to maximize CNC yield starting from bleached kraft eucalyptus pulp, using only COD yields, as they considered in all calculations these kinds of carbohydrate-derivatives, i.e., the inevitable presence of byproducts during the hydrolytic treatments. Hence, these authors sought and detected glucose, xylose, HMF, and furfural, in these acid hydrolysis environments of CNC generation. In this study, as well as in a former one [29], we detected less numerical values in COD CNC yield than in gravimetrical CNC yield, which is therefore consistent with the evidence provided by Wang et al. [35].

### 3.3. Morphological and Size Distributions of CNCs

**Figure 2:**
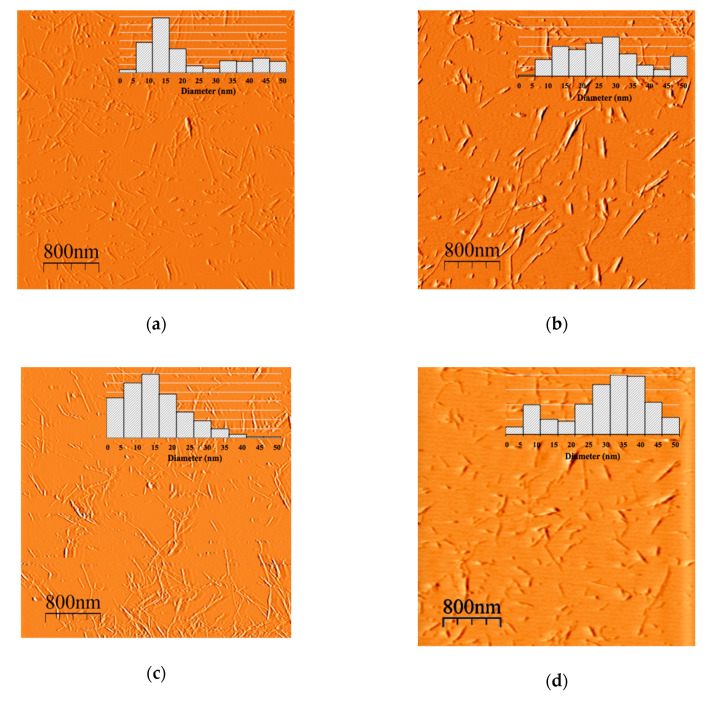

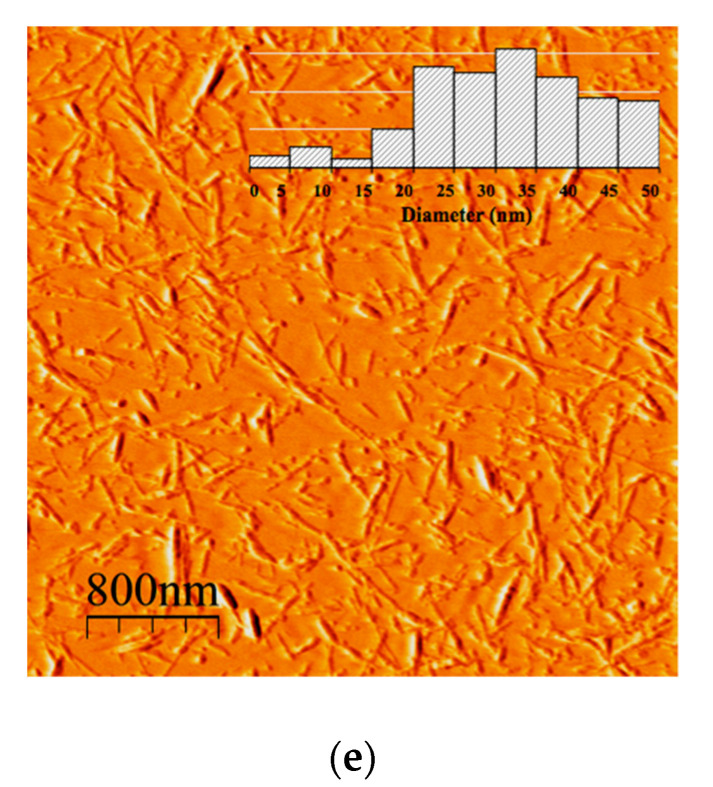
Atomic force microscope (AFM) images of the CNCs were analyzed. Each AFM image contains its graph of diameters size distribution. (**a**) Sample A; (**b**) sample B; (**c**) sample C; (**d**) sample D and (**e**) sample E.

The AFM images of the CNCs presented in Figure 2 indicate that all nanocrystals can be described as individualized rod-like particles. The obtained results are similar to the images previously reported in the literature [29,36,37]. In the five samples studied, the CNC suspensions remained stable and did not show any phase separation. Samples A and C, prepared using a high sulfuric acid concentration (64.8 wt%) had a smaller diameter size distribution than those obtained using a low sulfuric acid concentration (B and D: 44.1 wt% or E: 54.5 wt%). Different cellulose sources influence the morphology and size of the CNC obtained; however, within the same raw material, the morphological and size distribution characteristics depend directly on the hydrolysis conditions. The acid hydrolysis process is exceptionally aggressive; it is not possible to control the size of the produced nanocrystals [12].

### 3.4. Sulfur Content and Zeta Potential of CNCs

The sulfur content thus primarily reflects the surface charge of the crystals and is crucial to the characterization and understanding of material properties [38]. The value of sulfur content in these five CNC samples varied from 7.3 mg g^−1^ to 11.6 mg g^−1^ (Table 4); similar values have been reported in the literature [8,29]. It is noticeable that the sulfur content in samples A and C is higher than in the other CNC samples, this since sulfation is affected mainly by high acid concentration (64.8 wt%) in the hydrolysis process. 

The Zeta potential values varied between −21.3 mV and −28.9 mV; thus, CNC suspensions are stable without the tendency to agglomerate and precipitate. CNC suspensions commonly have values from −20 mV to −50 mV [39,40], indicating that the synthesized samples are at a lower limit than that reported in the literature.

### 3.5. FTIR Analysis

**Figure 3:**
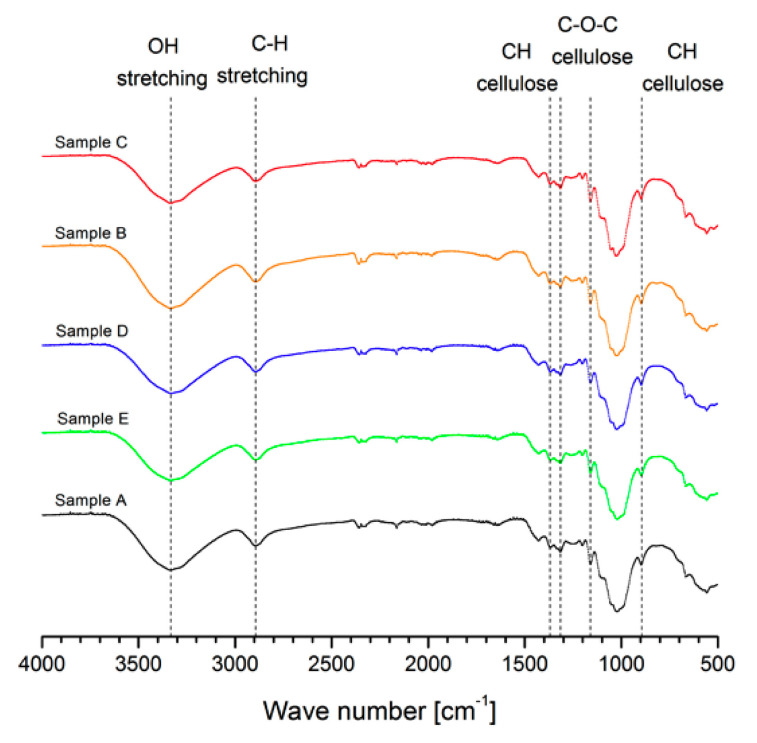
FTIR spectra for the five CNC samples selected.

The FTIR spectra of CNC samples are illustrated in Figure 3. The spectrum in the range 3400–3300 cm^−1^ is related to the stretching vibration of O-H groups having strong inter and intramolecular H-bonding in cellulose type I [22,23,29]. The stretching frequency at 2900 cm^−1^ was due to the symmetric C-H vibrations. The bond observed in the range of 1420–1430 cm^−1^ was attributed to the symmetric bending of CH_2_ and was also related to cellulose type I [41,42], the band at 1330–1380 cm^−1^ corresponded to the bending vibrations of the C-H and C-O groups of the polysaccharides [21,43], and the band at 1160 cm^−1^ is due to C-O-C asymmetric vibrations associated with cellulose I and cellulose II [29,43]. The band at 900 cm^−1^ indicates the typical structure of cellulose with β-glycoside bonds of glucose ring within the cellulose structure [23,44,45]. The FTIR spectra studied indicate that CNCs were successfully extracted from the hydrolysis treatment without secondary product formation.

### 3.6. X-ray Diffraction (XRD) Pattern

**Figure 4:**
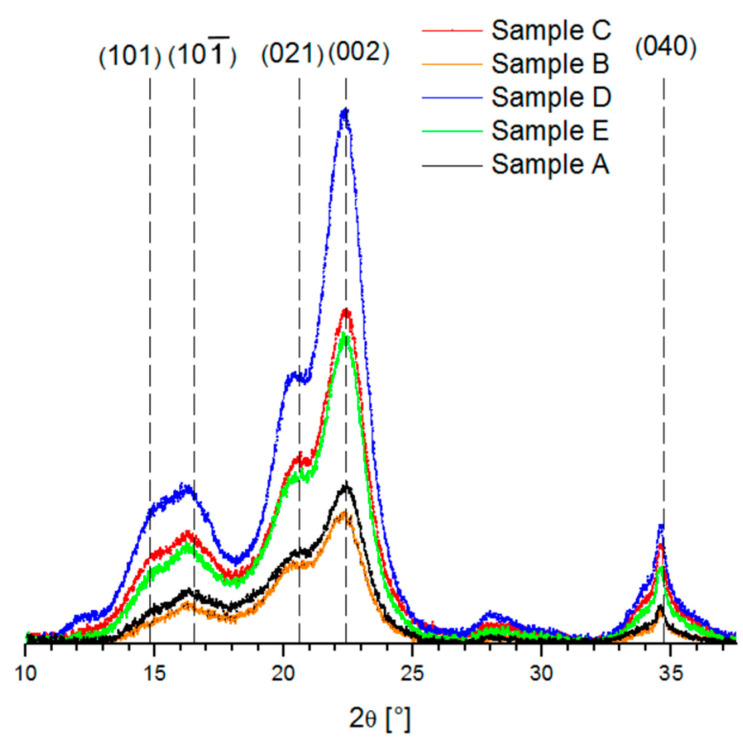
XRD spectra showing of the crystalline peaks associated with the five CNCs samples analyzed.

Figure 4 exhibits the XRD analysis for the five samples of CNC from kraft pulp. There were four peaks for CNCs at 2*θ* around of 15°, 16°, 22.5° and 34° attributed to the diffraction planes (1 0 1), (1 0 ∑1), (0 0 2) and (0 4 0), respectively, and are related to cellulose type I [22,45,46]. In addition, the angle at 20.6° attributed to the diffraction plane (0 2 1) is assigned to amorphous phases [29,46]. It is observed that for samples A and B, the peaks with Miller indexes (1 0 1) and (1 0 ∑1) become smaller, simultaneously the amount of cellulose in the amorphous state becomes larger, and therefore the crystallinity of the sample decreases.

As can be observed in Table 5, the crystallinity index value of samples varied between 66.1% and 81.9%, where D and C samples present higher crystallinity with 72.2% and 81.9%, respectively. The degree of crystallinity differs depending on the source of the raw materials, purification time of the sample, and hydrolysis conditions. There is a direct relationship between the crystallinity degree and hardness of the samples [23,47]. Besides, the crystallite size varied between 4.5 nm and 5.0 nm. The fractional variation in their interplanar distance (Δ*d*/*d*) of the CNC is used to analyze the strain and the micro-stresses in the crystalline planes [29,48], and for the CNC samples studied the values were in the range of 0.074 and 0.083.

### 3.7. Thermal Stability of CNCs

**Figure 5:**
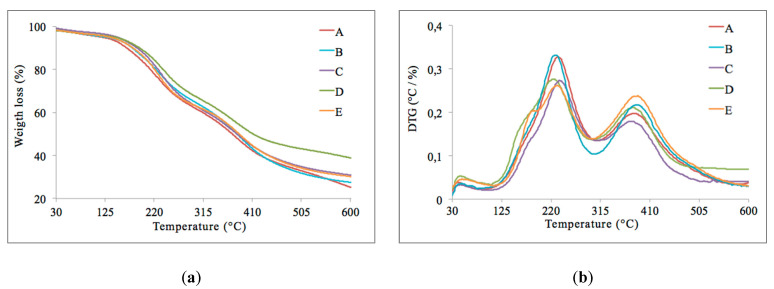
(**a**) Thermogravimetric (TG) curves and (**b**) derivative thermogravimetric (DTG) curves for the five samples of CNCs studied.

Thermogravimetric (TG) and derivative thermogravimetric (DTG) profiles obtained the five CNC samples are shown in Figure 5. There was a slight drop in the temperature near 100 °C in which indicates the evaporation of water. In all the CNC samples, two stages of CNCs degradation are observed. Similar antecedents have been reported by Kargarzadeh et al. [21] and Lin et al. [49]. The first stage presents a maximum peak of degradation temperature around 230 °C, and the second stage presents a maximum peak of degradation temperature above 350 °C (Figure 5b). The significant weight loss for CNCs was in the temperature range of 230–385 °C, and the samples underwent weight loss between 65% and 75% (based on dry weight). The presence of sulfate groups on the CNC surface might be a disadvantage for some applications since it favors its decomposition at lower temperatures [12,29,50].

### 3.8. Relationship between CNCs Features

**Figure 6:**
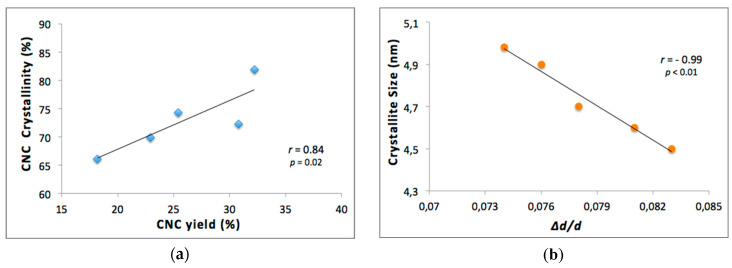

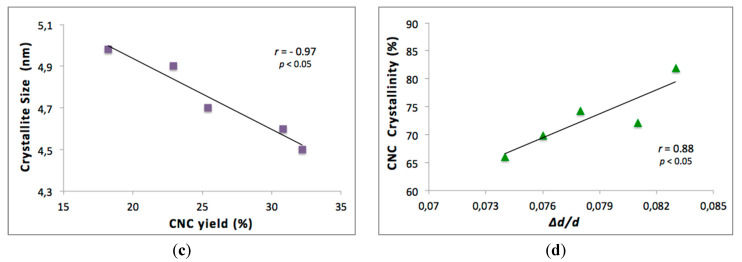
Regression lines and Pearson correlation between (**a**) CNC crystallinity vs. CNC yields; (**b**) Crystallite size vs. Δ*d*/*d*; (**c**) Crystallite size vs. CNC yield; (**d**) CNC Crystallinity vs. Δ*d*/*d*. The maximum standard error for CNC yield is 0.4%.

Synthetized CNCs have the potential to be used in various and diverse applications, so it is necessary to study their characteristics. Chen et al. [28] investigated CNCs from bleached kraft eucalyptus pulp. They indicated that for sulfuric acid concentrations above 58%, CNC yield should be inversely correlated to crystallinity because of the preferential hydrolysis of the more disordered regions. Authors have informed correlations between crystallinity and CNC yield were *r*^2^ = 0.39, *r*^2^ = 0.68 and *r*^2^ = 0.92 for acid concentrations of 58%, 62% and 64%, respectively. In our study, the CNC yield of the five samples analyzed (with acid concentrations between 44.1 wt% and 64.8 wt%) showed a significant Pearson correlation of *r* = 0.84; *p* = 0.02 (Figure 6a). Unlike the previously-mentioned study, a positive correlation was found in our data; this result can be attributed to various causes and the cellulose´s source might probably be the most important one. An investigation reported by Carrillo et al. [42] mentioned that celluloses obtained from different species of eucalyptus wood shows different crystalline structures and therefore influence their subsequent processing.

Riande et al. [51] mentioned that the properties of polymers materials depend on the crystalline structure and morphology, where “crystalline structure” refers to how the chains are packed in a particular conformation, giving rise to the three-dimensional structure. In contrast, “crystalline morphology” refers to the crystallites, their arrangement, and interconnection.

In CNC production, hydrolysis is a chemical process that increases mechanical stress on cellulose microfibrils. Therefore, in this process, CNCs are subjected to mechanical stress when separating from microfibrils, implying that Δ*d*/*d* increases, the latter being a measure of the microstrain of the crystal lattice that characterizes the average deformation within the crystallites of the CNCs. Therefore, with greater mechanical stress, Δ*d*/*d* increases and the crystallite size decreases. This observation can be verified in Figure 6b, where crystallites size versus fractional variation in their interplanar distance (Δ*d*/*d*) showed a significant but negative Pearson correlation of *r* = −0.99; *p* < 0.01. In literature, the importance of the potential variation between the distances of the crystalline planes in CNCs has been discussed, which affect properties in materials developed using these CNCs [52]. On the other hand, in the case of our data, the CNC yield may be related to the mechanical compression on the microfibrils during hydrolysis, and for this reason, the crystallite size decreases with increasing CNC yield (Figure 6c). In this sense, the increment in crystallinity is related to the average stress generated, since a higher CNC yield supposes a greater crystalline portion generated compared to the amorphous amount of cellulose obtained. Therefore, increasing yield is related to large mechanical stress on microfibrils, leading to an increase in the average deformation of crystallites of CNCs (Figure 6d).

**Figure 7:**
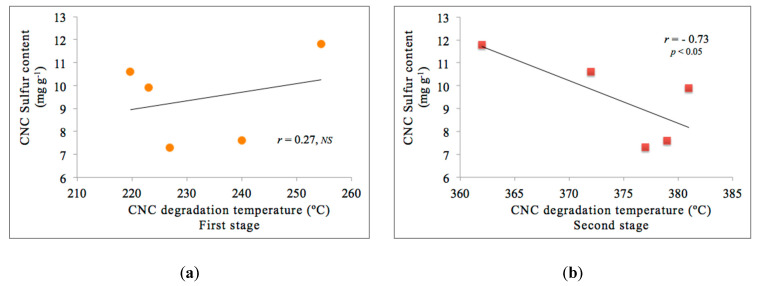
Regression lines and Pearson correlation between CNC sulfur content vs. CNC degradation temperature. (**a**) First stage degradation of DTG and (**b**) Second stage degradation of DTG. The maximum standard error for CNC sulfur content is 0.5 mg g^−1^ and for CNC degradation temperature in the first stage is 2 °C and the second stage is 1.5 °C.

Understanding the sulfate groups introduced on the CNC surface has become essential to evaluate the thermal degradation behavior of crystals prepared by sulfuric acid hydrolysis. According to data reported in this study, it is possible to observe two stages in CNCs thermal degradation (Figure 5b). The first stage corresponds to the degradation of the more accessible and sulfated amorphous regions. The second stage is related explicitly to the degradation of the unsulfated crystal [21,50], or also, to enhance access to the crystal interior due to the degradation of sulfated regions at the ends of crystals under harsh hydrolysis conditions [50]. Figure 7 shows the plots of the CNC sulfur content versus CNC degradation temperature in the first stage and the second stage from the profile analysis of DTG curves. In fact, a significant but negative Pearson correlation is observed in the second stage of CNCs degradation (*r*= −0.73; *p* < 0.05), confirming the degradation of the CNCs at this stage. This information is consistent with the thermal behavior of the CNC reported in the literature, since the presence of sulfate groups grafted on the CNC surface facilitates its decomposition, which means that at higher sulfur contents, the kinetics of degradation is accelerated. This correlation is not observed in the first stage of CNC degradation, Figure 7a (*r* = 0.27; *NS = not significant*).

Charged functional groups on the surface and zeta potential of CNCs are relevant properties in the manufacture of CNC based functional materials, as these properties dictate the ability of nanoparticles to disperse, aggregate, and/or interact with other materials [25,53]. Other studies have reported zeta potential values in CNCs similar to our work; therefore, it is expected that the individual CNC particles produced with similar functional groups exhibit similar stability and aggregation behavior. For instance, Ricón-Iglesias et al. [54] investigated the effect of CNCs surface charge and dielectric properties of poly (vinylidene fluoride) (PVDF) nanocomposites, indicating that CNCs with low values of zeta-potential (lower to −26.1) enhance the accumulated charge at the PVDF-CNC interface, improving the materials electric performance.

## 4. Conclusions

Research on CNCs and their applications as reinforcing materials has increased significantly in recent decades due to their exceptional properties. Several works aiming to optimize the yield and properties of CNCs have been published. This study discussed the physical and chemical characteristics of the CNC obtained from bleached kraft eucalyptus pulp by acid hydrolysis and showed the relationship among its different features. The top five CNC yields from an experiment design were analyzed, and the results obtained indicated that CNC possesse a morphology that can be described as stiff rod-like particles with average diameters less than 50 nm and different size distribution. Significant Pearson correlations were established between the crystallinity of the CNC versus CNC yield and versus Δ*d*/*d* and between the crystallite size versus the CNC yield and versus Δ*d*/*d*. In the derivative thermogravimetric (DTG) profile, two CNC degradation stages were observed, where the second stage shows a significant correlation between degradation and CNC sulfur content. In our analysis, the crystallographic parameters exhibited a correlation with the mechanical behavior of the CNCs, since the variation between the distances of the crystalline planes is related to the stress and deformation present on the CNC crystallites. The findings of this work will contribute to the knowledge of CNCs characteristics, and will provide relevant information for CNC-based industries and the processability of CNC materials.

## Figures and Tables

**Table 1:** Levels of the factors evaluated in the fractional factorial design, sample identification with the corresponding experimental conditions, and the main response evaluated in the yield of cellulose nanocrystals (CNC).

Factors Levels	Low (−1)	Central (0)	High (+1)
Kraft pulp (g 100 mL^−1^)	5.0	10.0	15.0
H_2_SO_4_ (wt*%*)	44.1	55.1	64.8
Time (min)	10.0	65.0	120.0
Temperature (°C)	40.0	60.0	80.0
Sonication time (min)	10.0	20.0	30.0

**Table 2:** Experimental conditions and the response of CNC yield.

Sample	Experimental Conditions ^1^					Response Yield (%)
	Kraft Pulp (g 100 ml^−1^)	H_2_SO_4_ (wt*%*)	Time of Reaction (min)	Temperature (°C)	Sonication Time (min)	
1	5 (−1)	44.1 (−1)	10 (−1)	40 (−1)	30 (+1)	2.9
2	15 (+1)	44.1 (−1)	10 (−1)	40 (−1)	10 (−1)	10.8
3	5 (−1)	64.8 (+1)	10 (−1)	40 (−1)	10 (−1)	3.2
4	15 (+1)	64.8 (+1)	10 (−1)	40 (−1)	30 (+1)	19.3
5	5 (−1)	44.1 (−1)	120 (+1)	40 (−1)	10 (−1)	11.5
6	15 (+1)	44.1 (−1)	120 (+1)	40 (−1)	30 (+1)	19.8
7	5 (−1)	64.8 (+1)	120 (+1)	40 (−1)	30 (+1)	15.4
8	15 (+1)	64.8 (+1)	120 (+1)	40 (−1)	10 (−1)	30.8
9	5 (−1)	44.1 (−1)	10 (−1)	80 (+1)	10 (−1)	9.5
10	15 (+1)	44.1 (−1)	10 (−1)	80 (+1)	30 (+1)	35.7
11	5 (−1)	64.8 (+1)	10 (−1)	80 (+1)	30 (+1)	50.7
12	15 (+1)	64.8 (+1)	10 (−1)	80 (+1)	10 (−1)	9.1
13	5 (−1)	44.1 (−1)	120 (+1)	80 (+1)	30 (+1)	53.9
14	15 (+1)	44.1 (−1)	120 (+1)	80 (+1)	10 (−1)	0
15	5 (−1)	64.8 (+1)	120 (+1)	80 (+1)	10 (−1)	0
16	15 (+1)	64.8 (+1)	120 (+1)	80 (+1)	30 (+1)	0
17	10 (0)	54.5 (0)	65 (0)	60 (0)	20 (0)	35.2
18	10 (0)	54.5 (0)	65 (0)	60 (0)	20 (0)	33.1
19	10 (0)	54.5 (0)	65 (0)	60 (0)	20 (0)	27.6

^1^ In parentheses, the orthogonal scale factors.

**Table 3:** CNCs yield of the five samples studied.

Sample Selected ^1^	CNC Samples	CNC Yield ^2^ (%)	CNC Yield by COD ^3^ (%)
8	A	30.8	18.2 (0.2)
10	B	35.7	22.9 (0.3)
11	C	50.7	30.8 (0.1)
13	D	53.9	32.2 (0.1)
17	E	35.2	23.4 (0.4)

^1^ Samples selected from Table 2 by yield. ^2^ Gravimetric yield (experimental design). ^3^ Values in parentheses correspond to the standard deviations.

**Table 4:** Sulfur content and Zeta potential values for CNC obtained from hydrolysis treatment.

CNC Sample ^1^	CNC Sulfur Content (mg g^−1^)	φz (mV)
A	10.6 (0.5)	−21.3 (1.2)
B	7.3 (0.3)	−24.2 (1.6)
C	11.6 (0.1)	−28.9 (1.2)
D	7.6 (0.5)	−25.8 (4.5)
E	9.9 (0.3)	−22.6 (0.6)

^1^ Values in parentheses correspond to the standard deviations.

**Table 5:** Crystallographic parameters of CNCs.

CNC Sample	Crystallinity Index (%)	Crystallite Size (nm)	Δ*d*/*d*
A	66.1	5.0	0.074
B	69.9	4.9	0.076
C	72.2	4.6	0.081
D	81.9	4.5	0.083
E	74.3	4.7	0.078
